# Pregnancy and delivery after myomectomy for large fibroids: utilization of trial of labor and obstetric outcomes in a single-center cohort study

**DOI:** 10.1007/s00404-026-08452-w

**Published:** 2026-05-12

**Authors:** Matan Mor, Or Gil, Noa Feldman, Maya Naor-Dovev, Neta Eisenberg, Noam Smorgick

**Affiliations:** 1 Department of Obstetrics and Gynecology, Shamir Medical Center (Assaf Harofeh), Zerifin, Israel; 2 Lis Maternity and Women’s Hospital, Tel Aviv Medical Center, Tel Aviv-Yafo, Israel; 3 Gray Faculty of Medical and Health Sciences, Tel Aviv University, Tel Aviv, Israel

**Keywords:** Myomectomy, Uterine rupture, Trial of labor after myomectomy (TOLAM), Cesarean delivery, Myomectomy-to-pregnancy interval

## Abstract

**Purpose:**

To quantify obstetric outcomes after prior myomectomy in a tertiary center and to explore whether operative and clinical characteristics are associated with uterine rupture.

**Methods:**

Single-center retrospective cohort of deliveries following laparotomic or laparoscopic myomectomy between August 2015 and January 2023. We extracted demographic, surgical, and obstetric data from electronic records, and analyzed only the first consecutive post-myomectomy pregnancy per patient.

**Results:**

Sixty-three women delivered after prior myomectomy. 75.5% underwent laparotomic myomectomy and the remainder laparoscopic. The mean largest myoma diameter was 9.6 ± 3.4 cm; 66.7% were intramural. Uterine cavity entry occurred in 30.4%. Trial of labor after myomectomy was attempted in four patients (6.3%) and was successful in three. Two patients had placenta accreta spectrum (3.2%), and two required transfusions for postpartum hemorrhage. One full-thickness uterine rupture was identified incidentally at a planned elective cesarean at 37.4 weeks (1/63; 1.6%) in an asymptomatic patient not in labor. A short myomectomy-to-pregnancy interval showed an association with rupture: < 3 months (OR 103.0, 95% CI 2.85–3728.2; *p* = 0.007) and < 6 months (OR 25.9, 95% CI 1.94–716.8; *p* = 0.037). No other variables reached statistical significance, although the analysis was underpowered to assess additional predictors.

**Conclusion:**

Uterine rupture after myomectomy was rare (1.6%) in our cohort and was associated with a short myomectomy-to-pregnancy interval. However, as this association is based on a single event, it should be interpreted with caution and considered hypothesis-generating. Despite the low absolute risk, and consistent with literature supporting TOLAM in selected patients, TOLAM was markedly underutilized in our center, highlighting a practice gap that may be addressed through more standardized counseling and delivery planning in tertiary settings.

## Introduction

Uterine leiomyomas are the most common benign gynecologic tumors. Myomectomy remains a widely used fertility-sparing treatment for symptoms, such as heavy menstrual bleeding, bulk-related discomfort, and subfertility. As more patients conceive after myomectomy, clinicians must address pregnancy-related risks and delivery planning. Key concerns include abnormal implantation (including scar implantation), placenta accreta spectrum disorders, and—although uncommon—the potential for uterine rupture. These considerations shape counseling and guide decisions regarding mode of delivery.

In clinical practice, the presence or absence of endometrial-cavity entry during myomectomy often drives mode-of-delivery recommendations, while other surgical variables receive less weight [1]. Although this approach reflects ACOG statements [2, 3], robust data validating it are scarce. Studies exploring additional predictors—such as energy modality, intramural tumor excision, and closure technique—have reported possible associations with rupture [4–7], yet results conflict and are limited by sample size and heterogeneity.

We aimed to estimate the risk of major obstetric complications after myomectomy in our institution and to examine whether clinical, operative, or myoma characteristics were associated with uterine rupture.

## Methods

A single-center retrospective study of patients who delivered at our center between August 2015 and January 2023.

### Study population

Patients with prior laparotomic or laparoscopic myomectomy who delivered during this period at our institution, Shamir Medical Center (Zerifin, Israel), a tertiary, university-affiliated hospital, were included in this study. Cases of vaginal or hysteroscopic myomectomy were excluded. Demographic, surgical, and obstetrical data were retrieved from computerized medical records.

Collected variables included baseline maternal and obstetric characteristics [age, prior obstetric history, and assisted reproductive technology (ART)]. We also extracted key surgical details (approach, myoma characteristics, cavity entry, and closure details) and pregnancy outcomes (mode of delivery, neonatal outcomes, and major complications including placenta accreta spectrum, postpartum hemorrhage with transfusion, and full-thickness uterine rupture).

Some of the patients conceived and delivered more than once. However, we included only the first consecutive post-myomectomy pregnancy in the analysis, in order to estimate more accurately the risk for uterine rupture, as the risk is highest in the first post-myomectomy pregnancy. To minimize missed early pregnancy events, we queried all cases coded as scar pregnancy and all uterine ruptures, and then manually reviewed records to identify events attributable to a prior myomectomy scar.

The primary outcome was full-thickness uterine rupture at any gestational age. Secondary outcomes included myomectomy scar pregnancy, placenta accreta spectrum, postpartum hemorrhage and blood transfusion, mode of delivery, and maternal and neonatal outcomes.

Laparoscopic myomectomy technique: Preoperatively, the patient routinely received 1 g of tranexamic acid and antibiotics. Vasopressin (20 IU in 1 mL diluted 1:100 with saline) was injected between the myoma capsule and the surrounding myometrium, and a horizontal incision was made just above the myoma using monopolar scissors. The myoma was pulled and enucleated by a laparoscopic tenaculum or a myoma screw. Hemostasis was obtained with judicious bipolar coagulation, with attention to minimizing thermal spread. Hysterotomy was closed with multilayer running sutures with braided thread (V-Loc™, Medtronic, Minneapolis, MN, USA or STRATAFIX™, Ethicon, Somerville, NJ, USA). The serosal layer was then closed with single absorbable sutures (Vicryl, Ethicon, Somerville, NJ, USA). All suturing procedures were performed intracorporeally. The myoma was extracted by in-bag cold morcellation through extension of the port incision. An anti-adhesion barrier was used routinely.

Laparotomic myomectomy was performed via a Pfannenstiel incision, using the same principles: vasopressin injection, incision over the myoma, traction and enucleation to the pseudo-capsule, limited coagulation, multilayer closure of the myometrium with VICRIL (Ethicon, Somerville, NJ) running suture and the serosal layer by single sutures.

All patients were counseled to avoid pregnancy for at least 3 months after surgery.

Patients who conceived after myomectomy were followed by our outpatient high-risk pregnancy team. There was no written protocol for the management of delivery after myomectomy in our institution. Delivery planning was individualized through shared decision-making in the third trimester. In practice, TOLAM is considered selectively when operative documentation is sufficiently detailed; otherwise, planned cesarean delivery is more commonly recommended, particularly in cases of uterine cavity entry**,** large intramural fibroid resection involving a substantial proportion of myometrial thickness**,** a short myomectomy-to-pregnancy interval**,** or uncertainty regarding the depth/extent of dissection or uterine closure details.

The study was approved by the local institutional review board (Approval No. 0265—25-ASF). Informed consent was waived due to the retrospective design of the study.

### Statistical analysis

Data were analyzed using IBM SPSS Statistics for Windows, Version 31.0 (IBM Corp., Armonk, NY, USA). Continuous variables were presented as mean and standard deviation, whereas categorical variables are presented as counts and percentages. Given the occurrence of a single uterine rupture event, conventional multivariable logistic regression was not feasible. Therefore, associations between uterine rupture and candidate risk factors were assessed using univariable Firth’s penalized logistic regression to obtain bias-reduced odds ratios (ORs) with 95% confidence intervals (CIs) and corresponding p values. Complete-case analysis was performed for each predictor according to data availability. A *p *value of < 0.05 was considered statistically significant.

## Results

There were no cases of myomectomy scar pregnancy or post-myomectomy early pregnancy uterine rupture during the study period [8, 9]. Following exclusion of hysteroscopic and vaginal procedures, 63 women with a history of myomectomy delivered during the study period. The mean maternal age was 36.8 ± 4.9 years. Most patients underwent myomectomy via laparotomy (75.5%), with the remainder treated laparoscopically. The mean diameter of the largest myoma removed was 9.6 ± 3.4 cm. By type, 66.7% of the largest myomas were intramural (FIGO 3–4), 25.6% subserosal (FIGO 5–7), and the remainder submucosal (FIGO 2). The anterior uterine wall was involved in 69.4% of cases, the posterior wall in 27.8%, and the fundus in 16.7%. On average, 1.9 ± 1.5 myomas were removed per patient, and the uterine cavity was entered in 30.4% of procedures (Table 1). No sarcomas were identified on histopathologic examination of the resected myomas.

**Table 1:** Baseline surgical and myoma characteristics of the cohort

			*N* = 63
Maternal age (years)			36.8 ± 4.9
Myomectomy surgical approach	Laparoscopy		24.50%
	Laparotomy		75.50%
Size of the largest myoma (cm)			9.6 ± 3.4
Largest myoma type*	Intramural		66.70%
	Subserosal		25.60%
	Submucosal		7.70%
Number of myomas removed			1.9 ± 1.5
Uterine wall involved by the largest myoma	Anterior		69.40%
	Posterior		27.80%
	Fundal		16.70%
Uterine cavity entry during myomectomy			30.40%

Continuous variables are presented as mean and standard deviation. Categorical variables are presented as percentages

*Percentages may exceed 100%, because some myomas involved more than one uterine wall

Among women with available data, 32.8% conceived by assisted reproductive technology (ART). The mean interval from myomectomy-to-conception was 34.8 ± 31.1 months; 11.3% of pregnancies occurred within 6 months of surgery and 3.8% within 3 months. Twin gestations accounted for 7.9% of pregnancies. A trial of labor was undertaken in 6.3% of patients and was successful in three of four cases. Overall, 93.7% (59/63) underwent planned cesarean delivery; among these women, 59.3% had “post-myomectomy state” as the primary indication. Significant adhesions were reported in 53.5% of cesarean deliveries (Table 2).

**Table 2:** Pregnancy, delivery characteristics, and maternal–neonatal outcomes in deliveries after prior myomectomy

Pregnancy characteristics	
ART	32.8% (19/58)
Twin gestation	7.9% (5/63)
Myomectomy-to-pregnancy interval (months)	34.8±31.1
Myomectomy-to-pregnancy interval < 3 months	3.8% (2/53)
Delivery characteristics	
Gestational age at delivery (weeks)	37.4±3
Mode of delivery	N=63
Trial of labor after myomectomy	6.3% (4/63)
Successful TOLAM	75.0% (3/4)
Intrapartum cesarean delivery	25.0% (1/4)
Elective cesarean delivery	93.7% (59/63)
Elective CS for post-myomectomy state as the primary indication	59.3% (35/59)
Elective CS for other indications	40.7% (24/59)
Maternal and neonatal outcomes	
Mean birthweight (gram)	3029.2±535.4
Apgar 1 <7	1.6%
Apgar 5 <7	1.6%
Umbilical artery pH	7.3±0.1
Adhesions during CS	53.5% (31/58)
Placenta accreta spectrum	3.2% (2/63)
Postpartum hemorrhage with transfusion of blood products	3.2% (2/63)
Uterine scar dehiscence	3.2% (2/63)
Uterine rupture	1.6% (1/63)

Continuous variables are presented as mean and standard deviation. Categorical variables are presented as percentages

*CS* cesarean section

Mean gestational age at delivery was 37.4 ± 3 weeks and mean birthweight was 3029.2 ± 535.4 g. One neonate (1.6%) had an Apgar score < 7 at both 1 and 5 min. Mean umbilical cord pH was 7.3 ± 0.1.

Placenta accreta spectrum was diagnosed in two patients (3.2%), and neither required hysterectomy. Two patients experienced postpartum hemorrhage and required blood products. Two additional patients were found to have uterine scar dehiscence at cesarean delivery; both underwent preterm cesarean section at 35.4 and 35.6 weeks, respectively, because of painful contractions in the setting of a prior myomectomy, suggesting uterine rupture. Maternal and neonatal outcomes were favorable in both cases, and no further intervention was required.

There were no cases of preterm uterine rupture. One patient was diagnosed intraoperatively with a full-thickness uterine rupture at the time of cesarean delivery, corresponding to a uterine rupture rate of 1.6%. She had previously undergone open myomectomy for secondary infertility, including removal of a 6 cm submucosal (FIGO type 2) myoma from the anterior wall which distorted the uterine cavity. The hysterotomy was performed with monopolar energy and bipolar coagulation; the uterine cavity was not entered. Closure was completed in multiple layers using 1-0 absorbable thread (Vicryl^®^, Ethicon, Somerville, NJ, USA), with a baseball serosal suture. A 1 cm subserosal myoma was also excised from the posterior wall. Although advised to avoid conception for at least 3 months, she conceived 9 weeks after surgery. The pregnancy course was otherwise unremarkable, and an elective cesarean delivery was scheduled at 37.4 weeks because of prior myomectomy and the short surgery-to-pregnancy interval. Preoperative ultrasound was normal. At surgery, a 2 cm full-layer rupture was identified at the prior myomectomy site. Delivery proceeded as planned, and both maternal and neonatal outcomes were favorable. The patient subsequently conceived again and delivered uneventfully by repeat cesarean section (not included in the present analysis).

**Fig. 1:**
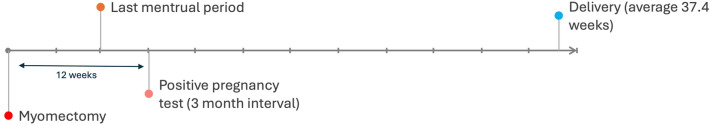
Timeline for myomectomy-to-pregnancy interval of 3 month

Univariable logistic regression, performed to explore potential predictors of uterine rupture, showed that a short myomectomy-to-pregnancy interval was associated with rupture. An interval < 3 months (Fig. 1) was significantly associated with uterine rupture (OR 103.0, 95% CI 2.85–3728.2; *p* = 0.007), and an interval < 6 months was also significant (OR 25.9, 95% CI 1.94–716.8; *p* = 0.037). No other evaluated clinical or surgical variables—including uterine cavity entry during myomectomy, twin gestation, surgical approach (laparoscopy vs laparotomy), and myoma location within the uterus—demonstrated a statistically significant association with rupture.

## Discussion

This study presents our data on pregnancies and deliveries after myomectomy. To mitigate pregnancy-related risks after myomectomy, we generally prioritize non-surgical management for patients of reproductive age and, when feasible, defer myomectomy until childbearing is complete. This practice is reflected in our cohort, in which the mean diameter of the largest resected myoma approached 10 cm, suggesting that smaller lesions were more often managed conservatively.

We found an association between a short myomectomy-to-pregnancy interval and uterine rupture. Uterine scar healing is a complex, multi-phase biologic process. It involves inflammatory mediators, extracellular matrix deposition, and progressive collagen remodeling that collectively contribute to scar maturation [10, 11]. In murine models, restoration of myometrial architecture may take months. Li et al. [12] reported complete recovery of the circular myometrium by 90 days post-surgery, whereas the longitudinal myometrium remained discontinuous at this time point. These experimental data support the biological plausibility that conception soon after myomectomy may occur before full scar maturation.

A short interpregnancy interval is an established risk factor for rupture after cesarean delivery and it is intuitively tempting to extrapolate this concept to myomectomy. Nevertheless, the available evidence is less robust. In an 11-year, population-based Korean study including 9890 pregnancies after myomectomy, the risk of uterine rupture was higher among women who conceived within the first postoperative year than among those who conceived during the second year (0.71% vs 0.35%). The risk then appeared to plateau at approximately 0.2% beyond two years after surgery [13].

Importantly, uterine rupture occurred only once in our cohort, and the study was underpowered to evaluate operative predictors. Therefore, non-significant findings for other surgical variables should be interpreted as absence of detectable association rather than evidence of no effect.

Studies report wide variation in trial of labor after myomectomy (TOLAM) rates. This likely reflects differences in institutional policies and clinician preferences, with reported rates ranging from 0 to 100% [14–18]. Despite this variability, studies with higher TOLAM uptake have generally reported favorable outcomes. This appears particularly true among patients who reach term gestation, with low rates of severe maternal complications [7, 14, 19]. Reported TOLAM success rates (approximately 75–93%) are comparable to those of TOLAC. In some series, TOLAM success rates are even higher than the 60–87% success rates commonly reported for TOLAC.

Our center has experience with vaginal birth after cesarean delivery (VBAC), reflecting a clinical environment familiar with managing trial of labor. However, this experience does not directly extend to trial of labor after myomectomy (TOLAM), for which data in our setting remain limited. Accordingly, our obstetric practice has been more conservative in this context, with TOLAM attempted in only 6.3% of the study population.

We believe that this discrepancy reflects lower institutional exposure to TOLAM rather than an evidence-based contraindication. Pregnancies after myomectomy are encountered less frequently, operative details may be less standardized, and delivery planning is often performed by teams not involved in the index procedure. In contrast, cesarean delivery is highly standardized, documentation is more uniform, and clinical teams have substantially greater experience counseling and managing trial of labor after cesarean.

In addition, although not strongly evidence-based, many obstetric units tend to avoid TOLAM when entry into the endometrial cavity is documented; this occurred in approximately 30% of cases in the present cohort and likely contributed to the conservative pattern observed. Another relevant factor is that most fibroids were relatively large and predominantly intramural, such that their resection involved a substantial proportion of uterine wall thickness, potentially increasing perceived risk. Finally, when the index myomectomy was performed outside our institution, operative reports often lacked key parameters (e.g., number and location of hysterotomies, depth and extent of dissection, cavity entry, and closure details), which may have further discouraged offering TOLAM.

The rupture was detected at term. Nevertheless**,** we classified it as an antepartum rupture, because the patient was asymptomatic, not in labor, and the defect was identified incidentally at elective cesarean delivery. The observed rupture rate in our cohort (1/63; 1.6%) is consistent with previously reported antepartum rupture rates. In the meta-analysis by Claeys et al., antepartum rupture occurred in 28 of 3685 pregnancies (0.76%) [7]. In a separate systematic review restricted to viable pregnancies, Gambacorti-Passerini et al. [6] reported an antepartum rupture rate of 1.52% (5/330).

We present an illustrative, exploratory projection based on published TOLAM success and rupture rates [6] to contextualize the potential impact of higher TOLAM uptake. This projection relies on simplifying assumptions and should be interpreted with caution.

In our cohort, only 4 women attempted TOLAM, while 59 had planned cesarean delivery, of whom 35 had “post-myomectomy state” as the primary indication. If these women had instead attempted TOLAM, assuming a 90.4% success rate, this would correspond to approximately 35 vaginal births and 4 intrapartum cesarean deliveries among the 39 women who might have been considered candidates for TOLAM based on available clinical information. The remaining 24 women would still have required planned cesarean delivery for other indications.

**Fig. 2:**
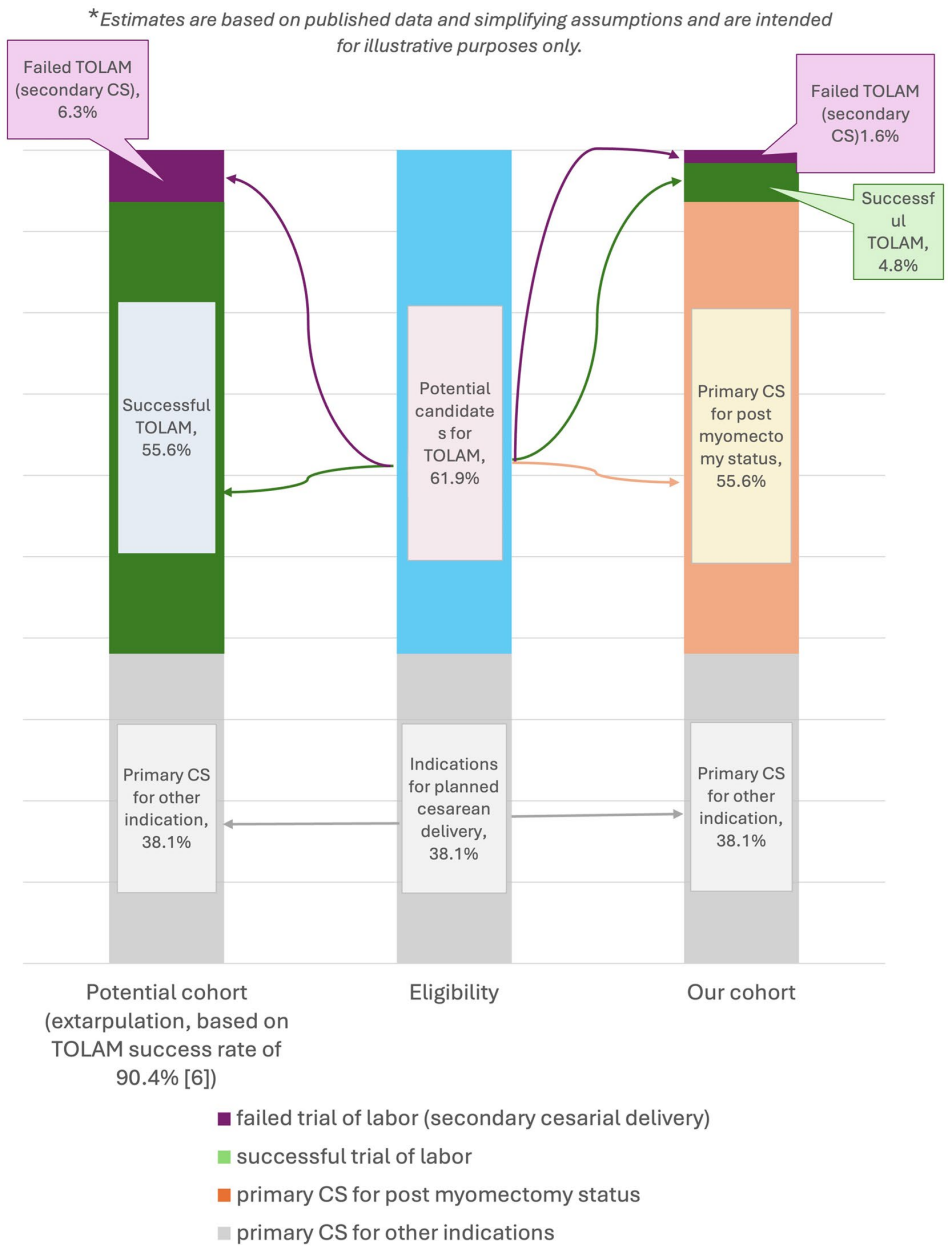
Observed delivery outcomes vs illustrative projection assuming TOLAM among potential candidates

Overall, the cesarean rate could decrease from 93.7% (59/63) to approximately 44% (≈28/63) (Fig. 2). Using a reported intrapartum rupture risk of 0.47% per TOLAM, the expected number of intrapartum ruptures would remain < 1 event (~ 0.18/39), corresponding to a number-needed-to-treat of ~ 213 elective cesareans to prevent one intrapartum rupture.

These estimates are illustrative and may over- or underestimate the true effect; they are intended to demonstrate potential magnitude rather than provide precise predictions.

Most reported ruptures after myomectomy occur antepartum and are, therefore, not prevented by avoiding labor. Term candidates can deliver in a tertiary center prepared to manage obstetric emergencies. In this setting, TOLAM may be considered for patients without specific high-risk features, and the planned mode of delivery should be finalized through shared decision-making with the patient. For patients who reach term after an uneventful pregnancy, published data suggest that the intrapartum rupture risk during TOLAM is low and broadly comparable to the widely accepted risk during TOLAC.

With only one uterine rupture event observed, the analysis had limited statistical power, and the findings should be interpreted cautiously. Larger studies with broader patient cohorts are needed to validate these results.

### Strengths and limitations

The main strengths of this study are that it was conducted in a single center, with a relatively consistent clinical practice and documentation. We included consecutive pregnancies after myomectomy to reflect real-world decision-making and to estimate the absolute risk of uterine rupture after the index procedure. Importantly, we used a strict clinical definition of uterine rupture and counted only full-thickness myometrial defects; uterine scar dehiscence found at cesarean section was not classified as rupture.

The main limitation is the retrospective design, with incomplete documentation of some surgical variables, which limits the ability to explore procedure-level predictors in detail. Detailed operative technique variables were not analyzed as predictors, because practice in our institution is largely standardized, resulting in limited variability. In addition, some index myomectomies were performed outside our center, and operative reports frequently lacked sufficient technical detail. Therefore, identifying predictors of uterine rupture based on surgical parameters was not feasible in this cohort.

Moreover, uterine rupture was very rare in our cohort (one case). Because of this, we could not perform reliable adjusted analyses, and any risk estimates are imprecise with wide confidence intervals. Importantly, the study was not powered to evaluate additional clinical or operative predictors, and non-significant findings should be interpreted as absence of detectable association rather than evidence of no effect.

Finally, since most patients were managed with planned cesarean delivery and only a small proportion underwent TOLAM, our cohort cannot be used to support strong conclusions regarding the safety of TOLAM versus planned cesarean delivery.

Future studies should be prospective and multicenter, with standardized recording of operative details and planned follow-up. Large registries or well-designed prospective cohorts may be more feasible than randomized trials and could identify predictors of rupture and outcomes with different delivery strategies.

## Conclusions

In this single-center cohort of consecutive pregnancies after laparotomic or laparoscopic myomectomy, we observed one case of uterine rupture, corresponding to a low absolute risk. A short myomectomy-to-pregnancy interval was associated with rupture in univariable analyses; however, as this finding is based on a single event with wide confidence intervals, it should be interpreted with caution and considered hypothesis-generating. Given the limited statistical power, the absence of statistically significant associations for other clinical and surgical variables should not be interpreted as evidence of no effect.

TOLAM was rarely attempted in our population, despite a substantially larger proportion of women who might have been considered candidates for trial of labor. This conservative approach likely reflects limited experience and inconsistent documentation, rather than clear contraindications. In tertiary centers prepared to manage obstetric emergencies, TOLAM may be considered more proactively for carefully selected term candidates, in line with published evidence and through shared decision-making. This should be accompanied by counseling to delay conception after myomectomy and efforts to improve standardized operative reporting and follow-up.

## Data Availability

The datasets are not publicly available due to patient privacy and institutional restrictions but are available from the corresponding author on reasonable request and subject to approval.

## Abbreviations

TOLAM: Trial of labor after myomectomy
TOLAC: Trial of labor after cesarean
VBAC: Vaginal birth after cesarean
ART: Assisted reproductive technology
CS: Cesarean section
OR: Odds ratio
CI: Confidence interval
PAS: Placenta accreta spectrum
